# Bone Metabolism Defects in Children With Idiopathic Hypercalciuria: An Update

**DOI:** 10.7759/cureus.82931

**Published:** 2025-04-24

**Authors:** Maria Pavlou, Anastasios Serbis, Maria Kostara, Anna Challa, Ekaterini Siomou

**Affiliations:** 1 Department of Pediatrics, University Hospital of Ioannina, Ioannina, GRC

**Keywords:** bone formation indices, bone mass density, bone resorption indices, children, idiopathic hypercalciuria

## Abstract

Idiopathic hypercalciuria (IH) in adults is considered to be the most common identifiable metabolic risk factor for calcium nephrolithiasis, also contributing to osteopenia and osteoporosis. Data on children and adolescents associating IH with bone metabolism show that up to one-third of such patients present with lower bone mineral density (BMD), increasing the risk of osteopenia, osteoporosis, and bone fractures in adulthood. Several factors, such as the degree of hypercalciuria and the presence of calcium urolithiasis, seem to affect the severity of bone metabolism abnormalities in children with IH. In order to follow these patients, BMD has traditionally been estimated by dual-energy X-ray absorptiometry (DXA) scan. In children, chronological age should be taken into account when measuring BMD, as well as weight, height, and BMI. In addition, biochemical bone turnover markers provide surrogate indices of bone turnover and complement the static measurements of BMD. They respond rapidly to changes in bone physiology, and their measurement can be repeated more frequently. However, since children’s bone mass increases constantly until after puberty, age, sex, and pubertal stage have to be taken into consideration when assessing these markers. In addition, relevant studies in children and adolescents have shown conflicting results. Regarding the management of patients with IH, identification and appropriate treatment are of great importance in order to prevent the formation of kidney stones, as well as to improve bone metabolism defects and decrease fracture risk. Such treatment measures include dietary interventions, potassium citrate supplementation and/or thiazide diuretics, and bisphosphonates in resistant cases. This review summarizes the latest data on bone metabolism defects in children and adolescents with IH, the possible pathomechanisms involved, the biochemical markers that could be used together with DXA to follow these patients, and the available treatment options.

## Introduction and background

Hypercalciuria in children is defined as the 24-hour urinary excretion of calcium over 4 mg (0.1 mmol)/kg of body weight [1] or an increased random or spot urine calcium/creatinine (UCa/UCr) ratio, with abnormal values varying with age [2,3]. The prevalence of hypercalciuria in children is 3.8-9.6% in population studies [4-7].

Hypercalciuria can be classified as either idiopathic or secondary [8]. Idiopathic hypercalciuria (IH) with normal serum calcium levels is the most common form of hypercalciuria. Its etiology is multifactorial, involving genetic, environmental, and dietary influences, and is mediated by various hormones including vitamin D, parathyroid hormone (PTH), calcitonin, thyroid hormones, sex hormones, growth hormone, insulin, glucocorticoids, and fibroblast growth factor-23, as well as interleukins and possibly other yet unidentified factors [9-13].

The incidence of IH in children is variable, ranging between 2.2 and 6.4% [7,14,15]. However, in a relatively recent study carried out in 2014 with children of European origin, the prevalence was found to be 17.7%, implying a possible increasing tendency [16].

Pak CY et al. in 1975 defined three forms of IH: intestinal (absorptive), due to intestinal calcium hyperabsorption; renal (fasting), due to tubular calcium leakage; and resorptive, due to excessive output of calcium from bone [16]. Bataille P et al., in 1998, based on further studies, modified this classification, taking into consideration the values of calcium, phosphorus, and PTH [17]. The following forms of IH and their associated mechanisms have been distinguished: Absorptive Hypercalciuria Type I is caused by elevated serum vitamin D levels or increased sensitivity to vitamin D and its metabolites, leading to chronic suppression of PTH secretion. PTH levels are typically normal or low. The fasting UCa/UCr ratio is not elevated. Absorptive Hypercalciuria Type II is a less severe form that generally responds to moderate dietary calcium restriction. Serum vitamin D and fasting UCa/UCr ratios are within normal limits, while PTH levels are normal to slightly elevated. Absorptive Hypercalciuria Type III is attributed to reduced renal phosphate reabsorption, which leads to hypophosphatemia and a compensatory increase in 1,25-dihydroxyvitamin D [1,25(OH)₂D]. This enhances secondary intestinal calcium absorption. PTH levels are normal or low, and the fasting UCa/UCr ratio is not elevated. Renal hypercalciuria results from primary renal calcium-wasting due to impaired tubular calcium reabsorption. This triggers secondary compensatory hyperparathyroidism and an increase in 1,25(OH)₂D levels, further enhancing intestinal calcium absorption. The fasting UCa/UCr ratio is elevated. Resorptive hypercalciuria is due to primary increased bone resorption, resulting in elevated calcium release into the bloodstream. PTH levels are normal or low, 1,25(OH)₂D levels are normal or elevated, and the fasting UCa/UCr ratio is elevated.

Given the close interplay among the GI tract, bones, and kidneys in maintaining calcium balance, IH appears to represent a continuous spectrum rather than distinct, isolated disorders. A primary defect in one of these systems often triggers compensatory adaptations in the others, for example, increased bone resorption in response to excessive renal calcium loss [18-22].

The clinical manifestations of pediatric IH include hematuria, renal colic, abdominal pain, UTI, and voiding dysfunction symptoms [23,24]. Between 5% and 57% of these children present with nephrocalcinosis or nephrolithiasis [25], and 20-38% are reported to have low bone mineral density (BMD), as estimated by dual-energy X-ray absorptiometry (DXA) [26-30]. The exact mechanisms have not yet been clarified, but several possible causes have been implicated in its pathogenesis [31]. More specifically, adult patients with hypercalciuria invariably demonstrate increased bone resorption, as assessed from osteoclastic activity and eroded bone surfaces. In addition, they manifest chronic mild parathyroid hormone elevation that stimulates bone resorption, leading to increased osteoclastic activity and bone resorption markers [32,33]. Also, patients with absorptive IH demonstrate reduced bone formation in bone biopsy. Bone resorption parameters are generally in the normal range but could be considered inappropriately high in the face of low BMD [32,34,35].

Absorptiometry and specific biochemical bone marker assessments have partly contributed to the clarification of the disease pathogenesis, especially in children. So far, most studies have shown normal longitudinal bone growth in most children with IH [31,36-40]. However, there are some studies showing significantly reduced final height in children with cofactors such as nephrocalcinosis [27,41] and hypocitraturia [42], suggesting further impact on bone metabolism in these patients.

Bone mass is determined by a balance between osteoblasts and osteoclasts that affects bone structure, degree of mineralization, and collagen and osteoid characteristics through bone turnover [43]. Any disturbance of this balance can lead to an increase or decrease in bone mass, with higher or lower BMD, respectively. Low BMD increases bone fragility and the risk of fractures [44]. Since bone mass reaches its peak density just after the second decade of life, the higher the bone mass achieved until early adulthood, the lower the risk for later fractures [45]. IH has been shown to be implicated in bone metabolism disturbances due to continuous urinary calcium loss, which can result in a negative calcium balance, affecting BMD and overall bone health, with increased risk of osteopenia, osteoporosis, and bone fractures in adult life [14,39,46,47].

This may explain why 20-38% of children with IH exhibit impaired bone metabolism and reduced BMD. The key question is the extent to which bone involvement occurs in this population, and how it can be accurately assessed, particularly in children and adolescents whose bones are still developing throughout puberty.

This review summarizes the latest findings on bone metabolism in children with IH, the role of biochemical markers, and current treatment strategies.

## Review

Materials and methods

Although this is a narrative review, the literature search was conducted in accordance with the Preferred Reporting Items for Systematic Reviews and Meta-Analyses (PRISMA) guidelines to ensure transparency and methodological rigor. A comprehensive search of the PubMed/MEDLINE database was performed to identify peer-reviewed articles published up to February 2025. The search strategy included the following keywords, used individually and in combination: “idiopathic hypercalciuria,” “hypercalciuria,” “bone mineral density,” “nephrolithiasis,” “urolithiasis,” “children,” “adolescents,” “calcium metabolism,” “bone metabolism markers,” and “growth.” Only articles written in English and focused on pediatric populations (children and adolescents) were considered for inclusion. Case reports, reviews, editorials, letters to the editor, and case-control studies were excluded. The selection process involved an initial screening based on titles and abstracts, followed by full-text review of the relevant studies. Additional pertinent articles were identified by reviewing the reference lists of the retrieved papers.

The initial search yielded 1,273 records; of these, 732 were excluded based on title and 268 after abstract screening. An additional 44 articles were excluded for being non-English. Manual screening of reference lists identified 41 additional relevant articles. Of the remaining 229 full-text articles, 199 were excluded for not meeting the inclusion criteria or lacking relevance to the review's objectives. In total, 71 articles were included in the final review.

Limitations of this review include the heterogeneity of study designs, populations, and outcome measures, which may affect the comparability and generalizability of findings. Furthermore, potential publication bias, such as the underreporting of negative or inconclusive results, cannot be excluded.

Pathophysiology of bone alterations in IH

The PTH/1,25(OH)₂D system helps in the fine-tuning of serum Ca concentration by regulating the synthesis and activity of transporters responsible for Ca transport among the three involved systems. A decrease in extracellular Ca concentration in IH should result in the immediate release of PTH to restore and conserve serum Ca levels by increasing renal Ca reabsorption and bone resorption.

In IH, PTH levels are typically low to normal. This is because IH is most often caused by increased intestinal Ca absorption, resulting in increased serum Ca levels and subsequently decreased PTH levels [48]. In a few cases of renal Ca leak hypercalciuria, there is an obligatory loss of Ca in the urine, which can lead to hypocalcemia and increased PTH levels. However, in the context of IH, PTH is not the primary driver of the condition. Instead, the focus is on the genetic or idiopathic factors that affect Ca reabsorption and excretion pathways [23,49,50]. PTH levels in most studies in children with IH have been found to be normal and not related to BMD, while at the same time, there was a negative correlation between hypercalciuria and BMD [13,26,27,29,51].

Similarly, 1,25(OH)₂D levels have been reported to be normal in children with IH [27,40] and in an animal model [52]. High sensitivity to 1,25(OH)₂D has been suggested from studies in rats, due to either increased intestinal vitamin D receptors (VDRs), leading to intestinal Ca hyperabsorption, or increased response of the VDRs to normal levels of 1,25(OH)₂D that was also expressed in bones [53-55]. Other investigators have reported increased calcitriol levels in children with IH, without finding any difference between the osteopenic and the normal BMD groups [26].

Regarding cytokines, IL-1, IL-6, and TNF are considered local mediators of bone resorption, as they affect the proliferation of osteoclasts [56]. Undifferentiated expression of IL-1α was found in one study in children with IH, of whom 38% had low BMD compared with controls [27]. Prostaglandin E₂ (PGE₂) is a known potent stimulator of bone resorption, and it can also inhibit osteoblastic collagen synthesis. Studies in children with IH have shown increased urine levels of PGE₂, although no correlation was found with BMD [26]. IL-13 inhibits the production of pro-inflammatory cytokines, including IL-6. In a study by Kusumi K et al., conducted in seven male adolescents with urolithiasis and low total body BMD z-scores compared to controls, a significant positive correlation was found between higher urinary IL-13 levels and total body BMD z-scores [57].

The consumption of Na and rapidly metabolized carbohydrates (glucose, sucrose, and ethanol) increases urinary Ca excretion due to reduced Ca and Mg tubular reabsorption, especially in patients with IH [58,59]. Increased protein intake causes mild chronic acidosis, which is counteracted by the release of Ca carbonate from the bones. This process leads to further bone resorption [60,61].

All the above show that bone alterations in IH are the result of a complex interplay between hormonal, dietary, and environmental factors that are still poorly understood.

Bone metabolism assessment

Bone metabolism is assessed by measuring BMD or biochemical bone turnover markers, and in some cases by biopsies, which are not usually performed in children.

Traditionally, BMD has been estimated by DXA [62,63]. In children, chronological age, weight, height, and BMI should all be considered when measuring BMD. “Low bone mass for chronologic age” is the preferred term describing low bone mineral content or BMD, which is defined as a Z-score equal to or less than -2.0 [64].

On the other hand, biochemical bone turnover markers provide surrogate indices of bone turnover and complement the static measurements of BMD. They respond rapidly to changes in bone physiology, and their measurement can be repeated more frequently. Thus, they may help in detecting disease or therapy effects earlier than changes in bone mass. However, since children’s bone mass increases constantly until after puberty, age, sex, and pubertal stage must be taken into consideration when assessing these markers [65].

The biochemical markers for bone formation are bone or total alkaline phosphatase (bALP or tALP), osteocalcin (OC), and maturation by-products of collagen type I (Table 1) [65]. These by-products are Procollagen type I N-terminal propeptide (PINP) and Procollagen type I C-terminal propeptide (PICP) [66]. The biochemical indices of bone resorption derive from degradation products of collagen type I or non-collagenous proteins of the underlying bone matrix. Measurement of deoxypyridinoline (DPD) and pyridinoline (PYD) levels in 24-hour urine collections has been used in the past [67]. Serum and urine levels of fragments of N-terminal cross-linking telopeptide of type I collagen (NTX-I) and of carboxy-terminal cross-linking telopeptide of type I collagen (CTX-I) are currently used as bone resorption indices with high specificity (Table 1) [68]. 

**Table 1:** Bone formation and resorption markers.

Bone Formation Markers	Bone Resorption Markers
Bone or total alkaline phosphatase (bALP/ALP) enzyme	Deoxypyridinoline (DPD) in 24-hour urine
Osteocalcin (OC)	Pyridinoline (PYD) in 24-hour urine
Procollagen type I N-terminal propeptide (PINP)	Urine hydroxyproline
Procollagen type I C-terminal propeptide (PICP)	Tartrate-resistant acid phosphatase (TRAP)
-	Serum and urine levels of carboxy-terminal cross-linking telopeptide of type I collagen (CTX-I)
-	Serum and urine levels of N-terminal cross-linking telopeptide of type I collagen (NTX-I)
-	Serum β-Crosslaps

β-Crosslaps is a specific and sensitive serum marker of bone resorption, which is a degradation fragment of mature CTX-I [69,70]. The β-Crosslaps/OC ratio can be used as a marker of bone metabolism, as it estimates bone resorption compared to bone formation and reflects the condition of bone metabolism. Arrabal-Polo MA et al. in 2012 proposed this ratio for the evaluation of bone metabolism in patients with recurrent Ca urolithiasis [71]. Tartrate-resistant acid phosphatase (TRAP) is a group of enzymes synthesized mainly in bone, spleen, and lungs, and is used as a bone resorption marker. The subform b of the isoenzyme 5 of TRAP (TRACP5b) is most specific to osteoclasts. TRACP5b is considered to reflect the number, but not necessarily the activity, of osteoclasts.

In 2017, the International Foundation for Osteoporosis and the International Federation of Clinical Chemistry Bone Marker Standards Working Group identified serum PINP and CTX-I levels as reference markers for bone formation and resorption, respectively, for predicting fracture risk and monitoring osteoporosis treatment [69,72].

Evaluation of BMD in pediatric IH

Bone loss can be the result of either decreased bone formation, increased bone resorption, or an imbalance between the two. Investigators report that decreased BMD is found in 20-38% of children with IH [16,26,27,29,30,36,73,74-76].

This decreased BMD raises the question of whether osteoporosis identified in adult patients with IH has its roots in childhood [77-79]. The variability in incidence could be explained by differences in age and the presence or absence of urolithiasis, hypocitraturia (IHC), or hyperuricosuria in children across different studies.

Specifically, in a study of children with IH, BMD was found to be low, and even lower in the nephrolithiasis group [14]. Similarly, Artemiuk I et al. found low BMD in children with IH, but children with concomitant nephrolithiasis had even lower BMD (Table 2) [76]. Hence, nephrolithiasis in IH appears to further impair bone metabolism, as has been described in adult patients [80-82]. The few available studies in children reported that correction of hypercalciuria improves bone density. Penido MG et al., in a study of 80 children with IH followed for six years, found that BMD Z-scores were decreased in patients and improved after treatment with potassium citrate or potassium citrate and thiazides (Table 2) [39].

**Table 2:** Studies focused on BMD assessment in children with IH. BMD: Bone Mineral Density; PTH: Parathyroid Hormone; OC: Osteocalcin; iPTH: Intact Parathyroid Hormone; IH: Idiopathic Hypercalciuria; 25(OH)D₂: 25-hydroxyvitamin D₂; 1,25(OH)₂D₃: 1,25-dihydroxyvitamin D₃ (Calcitriol); 25OHD₃: 25-hydroxyvitamin D₃; DXA: Dual-energy X-ray Absorptiometry; UCa: Urinary Calcium; UCre: Urinary Creatinine; Ca/citrate: Calcium to Citrate Ratio in Urine; TRAP: Tartrate-Resistant Acid Phosphatase; DPD/Cre: Deoxypyridinoline to Creatinine Ratio; ALP: Alkaline Phosphatase; β-Crosslaps: Beta-Crosslaps (fragment of type I collagen used as a marker of bone resorption); eGFR: Estimated Glomerular Filtration Rate.

Study	Type of Study	Subject (Number)	BMD	Height	Presence of Urolithiasis	Other Findings
Escribano J, et al. (2014) [15]	Prospective	31 IH children and 135 controls	IH group had borderline lower whole body BMD compared to controls; BMD Z-scores were also lower	Undifferentiated height	None	IH is associated with lower BMD Z-scores and osteopenia.
García-Nieto V, et al. (1997) [26]	Prospective	73 children with IH and 57 controls	22/73 patients had low BMD	2/73 had short stature	20/73	
Freundlich M, et al. (2002) [27]	Prospective	21 IH children and their mothers	8/21 children had low BMD; 7/21 mothers had low BMD	Normal height	7/21	
Penido MG, et al. (2003) [28]	Prospective	88 children with IH and 29 controls	35% of patients had reduced BMD compared to controls	Undifferentiated height	49/88	Positive correlations between iPTH, OC, and BMD Z-score.
Penido MG, et al. (2012) [28]	Retrospective	80 children with IH before and after treatment for 6 years	Low BMD Z-score before treatment; increase in BMD after treatment	Undifferentiated height before and after treatment	43/80 had urolithiasis	A beneficial effect of treatment was observed in these patients.
Skalová S, et al. (2005) [29]	Prospective	15 children with IH	40% had BMD Z-scores between –1 SD and –2 SD; 20% had Z-score < –2 SD	Normal	2 had urolithiasis; 1 had nephrocalcinosis	
Schwaderer AL, et al. (2008) [30]	Retrospective	110 children (61 IH, 37 nephrolithiasis, 12 excluded); Urolithiasis group included 17 with hypercalciuria, 4 with hypocitraturia, 2 with oxaluria, and 14 mixed	25.5% had Z-score < –1	No difference between low and normal BMD groups	37/110 had nephrolithiasis	Low BMD group had a higher body mass index (BMI) Z-score and a higher male-to-female ratio.
Polito C, et al. (2003) [36]	Retrospective	26 children with IH (Group 1: 9 with hyperuricosuria, Group 2: 17 without)	3/26 children with IH had BMD Z-score < –1 (all from group 1)	Normal	Without urolithiasis	Children with long-standing IH and hyperuricosuria may be at risk for osteopenia.
Penido MG, et al. (2021) [39]	Retrospective	40 IH children: after an initial 4-month dietary modification, 2-month potassium citrate treatment; 1st DXA performed in 9/40 with normocalciuria (Group G1, continued K-citrate), 31/40 still hypercalciuric (Group G2, added thiazides); 2nd DXA after 1 year	1st DXA: G1 and G2 groups had lumbar spine (L1–L4) BMD Z-scores < –1. 2nd DXA: G1 changed from –1.3 to –1.6 (p = 0.16), not statistically significant; G2 improved from –1.7 to –1.4 (p = 0.04), statistically significant.	Normal	Urolithiasis not reported; no child had nephrocalcinosis	PTH and ALP levels were normal. BMI Z-score was undifferentiated between Group G1 and Group G2.
Pérez-Suárez G, et al. (2021) [40]	Longitudinal review	34 IH patients: 1st DXA (BMD1) at 10.5 ± 2.7 years, 2nd DXA (BMD2) at 14.5 ± 2.7 years, 3rd DXA (BMD3) at 28.3 ± 2.9 years	Slightly increased BMD between z-BMD3 and z-BMD1 (p = 0.001) and between z-BMD3 and z-BMD2 (p = 0.016); positive correlation between BMI1 and BMD1 (p = 0.01), and between BMI2 and both BMD2 (p = 0.04) and z-BMD2 (p = 0.03). In 20/34 with z-BMD2 < –1 (p = 0.03); in 18/34 with z-BMD3 < –1 (p = 0.006 and p = 0.01). Over time, z-BMD increased in both men and women, but the increase was statistically significant only in women. Comparison between men and women showed no significant differences in z-BMD1 or z-BMD2, but a significant difference for z-BMD3 (p = 0.036).	Normal height	9/34 had lithiasis at diagnosis; 13/34 had lithiasis in adulthood; none had nephrocalcinosis	Calcium levels were significantly higher in BMD1 than BMD3; iPTH levels were significantly lower in BMD1 than BMD3. UCa/UCre > 0.20 mg/mg in: • 100% of patients at diagnosis • 52.9% at BMD1 • 26.5% at BMD2 • 23.5% at BMD3 Urine Ca/citrate > 0.33 mg/mg decreased from: • 53.6% at diagnosis • 40.6% at BMD1 • 28.6% at BMD2 • Significantly higher in adulthood (70%) Osteocalcin levels were normal in: • 15/34 patients at BMD1 • 13/34 patients at BMD2 TRAP levels were normal in: • 14/34 patients at BMD1 • 13/34 patients at BMD2 DPD/Cre was normal in: • 14/34 patients at BMD1 • 13/34 patients at BMD2 Calcitriol levels were normal in 32/34 patients at BMD1. A negative correlation was found: • Between BMD1 and TRAP (p = 0.036) • Between BMD2 and DPD/Cre ratio (p = 0.03) In 3/8 patients with hypercalciuria in adulthood and citrate/cre < 250 mg/g, z-BMD1 was higher than BMD2. Urine citrate levels were undifferentiated between adults with and without hypercalciuria. In adulthood: • 3/34 patients had eGFR between 80 and 90 mL/min/1.73 m² • 4/34 had hypophosphatemia (2 with normocalciuria, 2 with hypercalciuria) Mean calcidiol level was 24.5 ± 7.8 ng/mL (7/34 had < 20 ng/mL; among them, 3 had z-BMD < –1; 3 had hypercalciuria; 1 patient had both). In adulthood, ALP levels and β-Crosslaps/creatinine ratio were normal. No correlation was found between these markers and BMD3.
Penido MG, et al. (2006) [42]	Prospective	88 children with IH (Group 1: 44 with hypocitraturia, Group 2: 44 without); Group 3: 29 controls	Children with IH had lower BMD compared with controls; those with hypocitraturia had even lower BMD than those without	Height was lower in children with IH and even lower in those with IH and hypocitraturia	Not reported	
García-Nieto V, et al. (2003) [74]	Prospective	40 IH girls and their mothers; 19 healthy girls and 17 healthy women as controls	48% of girls had low Z-scores	Not reported	Not reported	
Artemiuk I, et al. (2015) [76]	Prospective	31 IH children on a free diet with normal calcium intake	8/31 had low BMD Z-scores	Not reported	8/31	22/31 had 25OHD₃ levels < 20 ng/mL.
Perrone HC, et al. (1992) [82]	Prospective	20 children with absorptive IH	4/20 had low BMD	Not reported	Not reported	No correlation of Gla-protein, PTH, 25(OH)D₂, and 1,25(OH)₂D₃ between patient groups.
Stapleton FB, et al. (1989) [83]	Prospective	76 IH children and a control group	No difference between groups	Undifferentiated height	Not reported	No correlation was found between BMD, PTH, and osteocalcin.

It seems that by treating hypercalciuria, decreased bone density also improves. Perrone HC et al., in 1992, in a group of children with absorptive IH, observed that those put on a low-calcium diet (400-500 mg/day), apart from reducing hypercalciuria, also improved their lumbar spine BMD, while those on a free diet showed no improvement in BMD [82]. These findings appeared surprising, as dietary calcium restriction can lead to a secondary increase in PTH production and thus bone resorption, especially in growing children. In addition, an age dependence has been reported in untreated children with IH, since a negative correlation was observed between BMD and age, suggesting progressive bone loss [26].

The co-existence of hyperuricosuria is also considered a contributing factor to bone loss in IH. García-Nieto V et al. found low BMD in 30% of children with IH who also had hypocitraturia and hyperuricosuria [26]. Similarly, Penido MG et al. reported that the coexistence of idiopathic hypocitraturia in children with IH further negatively affects BMD in the absence of metabolic acidosis [42]. Since the bulk of citrate resides in mineralized tissues, in hypocitraturia, citrate is released from the bone tissue to restore the deficit. This occurs during the bone resorption phase together with calcium, thus contributing to a reduction in BMD and further worsening of hypercalciuria. Polito C et al., in a study of children with IH without nephrocalcinosis, found reduced lumbar spine BMD, and even lower values in those with concomitant hyperuricosuria [36].

Genetic predisposition is another factor under investigation. Freundlich M et al. measured BMD in children with IH and their asymptomatic mothers and found lower BMD in 38% of children and 33% of mothers. Among the asymptomatic mothers, 25% presented with hypercalciuria. In addition, children of osteopenic mothers had significantly lower lumbar BMD Z-scores compared to those with mothers with normal BMD [27]. In another study involving girls and their mothers with IH, a BMD Z-score < -1 was found in 42.5% of girls and 47.5% of mothers. BMD was significantly lower in girls whose mothers had osteopenia than in those whose mothers had normal BMD [73]. The findings of these studies imply a genetic predisposition to IH [20].

Evaluation of biochemical bone markers in pediatric IH

In the limited number of studies in children with IH, mainly ALP and OC have been assessed as bone formation markers. As bone resorption markers, urine DPD and PYD, urine NTX-I, serum β-Crosslaps, TRAP, and urine hydroxyproline were used (Table 3).

**Table 3:** Studies focused on bone formation and resorption markers in children with IH. BMD: Bone Mineral Density; IL-1α: Interleukin-1 alpha; Z-score: Standard Deviation Score; kB (in sRANKL): Kappa-B (a subunit of the NF-κB protein complex); sRANKL: Soluble Receptor Activator of Nuclear Factor Kappa-B Ligand.

Study	Type of Study	Subject (Number)	Bone Formation Markers	Bone Resorption Markers	BMD	Height	Presence of Urolithiasis	Other Findings
García-Nieto V, et al. (1997) [26]	Prospective	73 children with IH and 57 controls	Increased OC in patients compared to controls; no difference in OC between low and normal BMD patients	No difference in tartrate-resistant acid phosphatase and urine hydroxyproline between low and normal BMD patients	22/73 had low BMD	2/73 had short stature	20/73 had urolithiasis; 2/73 had nephrocalcinosis	–
Freundlich M, et al. (2002) [27]	Prospective	21 IH children and their asymptomatic mothers	Not evaluated	No difference in pyridinoline and deoxypyridinoline levels	8/21 children had low BMD; 7/21 mothers had low BMD	Normal height	7/21 had urolithiasis	Undifferentiated expression of IL-1α in blood cells
Penido MG, et al. (2003) [28]	Prospective	88 children with IH (32/88 with hyperuricosuria) and 29 controls	Increased ALP and a trend toward increased OC in patients compared to controls	Increased NTX-I/creatinine; trend toward increased pyridinoline; no difference in deoxypyridinoline	31/88 had reduced BMD	No significant difference in height between patient groups	49/88 had urolithiasis	–
Polito C, et al. (2003) [36]	Retrospective	26 children with IH (Group 1: 9 with hyperuricosuria; Group 2: 17 without), followed for 4–13 years	Normal ALP	Not evaluated	3/9 in Group 1 had BMD Z-score < –1	Normal height	None	–
Penido MG, et al. (2006) [42]	Prospective	88 children with IH (Group 1: 44 with hypocitraturia; Group 2: 44 without hypocitraturia); Group 3: 29 controls	Higher ALP in both patient groups compared to controls	Higher N-telopeptide levels in both patient groups compared to controls	Group 1 had lower BMD than Groups 2 and 3	Height was lower in Group 1 than in Groups 2 and 3; height was lower in Group 2 than in Group 3	Not reported	–
Pavlou M, et al. (2018) [38]	Prospective	50 children with IH at diagnosis and after 3-month dietary intervention; 50 controls	No difference in ALP and OC between patients and controls; values remained unchanged after 3 months in patients	β-Crosslaps and the β-Crosslaps/OC ratio were higher in patients at diagnosis compared to controls; β-Crosslaps showed a trend to decrease after 3 months	Not evaluated	No significant difference in height	11 had urolithiasis; 6 had nephrocalcinosis	No difference in osteoprotegerin and soluble receptor activator of nuclear factor kappa-B ligand (sRANKL) system

Measurements of both formation and resorption markers could reflect the rate of bone turnover. However, the results of studies on these markers are inconsistent, possibly due to differences in study populations.

Normal ALP values were reported in children with IH without nephrocalcinosis/urolithiasis and with normal height, and a BMD Z-score < -1 in only 11.5% (Table 3) [36].

The authors interpreted the results as indicative of normal bone formation. In a study comprising 40 children with IH and reduced BMD but without nephrolithiasis, height and ALP were found to be normal at diagnosis and during the one-year treatment [39]. In a study of children with IH and mild Ca urolithiasis, ALP and OC levels were found to be normal both at the time of diagnosis and after three months of dietary intervention, and remained undifferentiated despite a significant decrease in calciuria (Table 3), suggesting normal bone formation at the time of diagnosis [38]. In children with severe symptoms of IH, such as urolithiasis in more than half (56%), ALP was found to be increased irrespective of the presence or absence of hypocitraturia (Table 3) [42]. Perez-Suarez G et al., in 34 children with nephrolithiasis and reduced BMD, found normal OC levels at diagnosis and during re-evaluation after a median time of 4 years [40].

In addition, studies of bone resorption markers presented controversial results, even if some markers, such as crosslink peptides, have been reported to be increased in most studies (Table 3) [42]. Similarly, in our study, increased serum β-Crosslaps levels were found, with a trend toward decrease after three months of diet and reduction in calciuria. Also, the β-Crosslaps/OC ratio was higher in the patient group at the time of diagnosis compared to controls, indicating increased bone resorption activity [38]. García Nieto VM et al. reported significantly higher values of the CrossLaps/Cr ratios in children with IH, irrespective of osteopenia [80]. In an earlier study by Penido MG et al., NTX-I levels were also negatively correlated with BMD corrected for volume.

Urine hydroxyproline was reported not to differ between osteopenic and non-osteopenic children with IH [26]. Concerning PYD and DPD in children with IH, they do not differ from controls in most studies (Table 3). In another study, undifferentiated levels of PYD and DPD were found in children, despite low BMD in 38% of them compared to controls [27]. Similarly, in the study by Penido MG et al., urine PYD and DPD levels were undifferentiated in patients, but PYD alone had a negative correlation with lumbar spine BMD. García-Nieto V et al. reported higher values of DPD/Cr in children with IH, irrespective of the presence of osteopenia [80]. In a study by Perez-Suarez G et al. involving 34 children with nephrolithiasis and reduced BMD, normal levels of TRAP and urinary DPD/Cr were observed both at diagnosis and at re-evaluation after a median follow-up of four years. A negative correlation was identified between bone turnover markers and BMD at both time points. Notably, BMD remained unchanged over time, findings that are consistent with persistently reduced bone density [40].

Studies have also shown that correction of hypercalciuria seems to reverse adverse effects on bone metabolism [27,38,39,76,81-84]. Perrone HC et al., in an early prospective study of children with absorptive hypercalciuria, showed that dietary restriction led to improvement in lumbar spine BMD in those treated compared with those untreated [82]. More recently, Freundlich M and Alon US also related the reduction in Ca excretion with the concomitant improvement in BMD [81]. Penido MG et al., in two studies in 2012 and 2021, reported an increase in BMD after appropriate treatment [37,39]. Similarly, in our study, the increased β-Crosslaps levels showed a trend to decrease after 3 months of dietary modification [38].

From the few previous studies in children with IH, normal height has been reported, especially in those without nephrocalcinosis [37,39,40,83,85] (Tables 2-3). A decreased height was found in children who had co-factors that could play a role together with IH and reduced BMD. Hypocitraturia with increased bone markers was reported in one [42]; increased 1,25(OH)₂D and urine PGE₂ in another (Table 2) [27]; while the subjects of a third had nephrocalcinosis [41].

Genetics

IH-associated genes encode cellular and growth factors that may be involved in bone metabolism, collagenous or non-collagenous products of the bone matrix, calciotropic hormones and their receptors, or factors affecting calcium salt precipitation.

Recently, polymorphisms in some genes that encode certain proteins involved in tubular calcium or phosphate reabsorption (CASR, SLC34A1, SLC34A4, CLDN14, CaSR, TRPV6, TRPV5), or in the prevention of precipitation of calcium salts (CaSR, MGP, OPN, PLAU, UMOD), have been associated with IH and kidney stones [12,22,49,85-87].

Treatment of IH and bone metabolism

The first line of management in children with IH is a diet that can ameliorate both hypercalciuria and hypocitraturia, as it also helps protect against stone formation [25,87]. Dietary recommendations include increased fluid intake, adequate calcium and protein intake, salt restriction, and increased potassium citrate intake [88].

If dietary and activity management fails, pharmacological therapy is initiated with potassium citrate supplementation and/or thiazide diuretics. In children with IH, administration of citrate salts reduces hypercalciuria and improves bone mass [37,87]. In a retrospective study, after one year of treatment in children with IH, an effect was reported only for combined thiazide and citrate therapy, while citrate monotherapy failed to increase the DXA Z-score of the lumbar spine. BMI was undifferentiated between the two groups (Table 2) [39]. However, data regarding the effect of thiazides on BMD in children with IH are contradictory [37,89,90].

Ferre N et al., in a 2022 review article, summarized that thiazides may improve BMD in patients with recurrent stones and hypercalciuria, although the long-term effect of their use remains unclear. They also reported uncertainty about whether diuretic use is associated with a higher incidence of serious adverse events compared with bisphosphonate therapy (e.g., alendronate) [90-91].

Orthophosphates are recommended for the minority of patients with renal phosphate leak hypercalciuria or those who do not respond to thiazides [22].

Finally, if all these treatments fail to increase BMD, especially in children with concomitant nephrolithiasis, bisphosphonates can be initiated [81,92]. Bisphosphonates block excessive bone resorption and improve BMD and some resorption markers. Heilberg IP et al. demonstrated that in young male patients with hypercalciuria and osteopenia, treatment with the bisphosphonate etidronate led to improvement in BMD [92]. Freundlich M and Alon US, in adolescents with persistent hypercalciuria and reduced BMD, found that treatment with appropriate diet, thiazides, and citrate salts had no effect on low BMD or the elevated urine NTX/Cr marker. On the contrary, the initiation of alendronate therapy increased their lumbar spine and femoral neck BMD Z-scores, while also reducing calciuria and decreasing the resorption marker N-telopeptides excretion [81].

Bisphosphonates are widely used to treat osteoporosis in children with chronic bone diseases and have generally been shown to be effective in improving bone density [93]. However, there are treatment-related adverse events, such as hypocalcemia, acute phase reactions, renal injury, osteonecrosis of the jaw [94], and more rarely, iritis, atypical femoral fractures (with long-term use), teratogenic effects, esophagitis from oral bisphosphonate use, and delayed bone healing in Osteogenesis Imperfecta. These must be carefully considered [93]. Duration of therapy depends on the improvement in BMD. Careful monitoring and management of potential side effects are essential [95].

In 2009, García-Nieto V et al. [80] evaluated the BMD of 104 children with IH on two occasions, without drug treatment. There were no differences in calciuria, citraturia, or age at the time of the two bone densitometries. A trend toward spontaneous improvement in BMD was observed, which was associated with increased body mass. The authors concluded that, except in the case of fractures, initial drug therapy with bisphosphonates or thiazides is not indicated [80].

Long-term outcome and prognosis

Bone health is established in childhood, as peak bone mass is usually achieved by age 20 [45]. Some authors believe that hypercalciuria is not a disease but rather the physiology of individuals who fall at one extreme of a continuous spectrum of UCa excretion rates [12]. However, if this spectrum of high calcium excretion leads to harmful consequences and diseases in adulthood, it could be considered a disease in pediatric patients. In most cases, it is simply a benign metabolic abnormality and should be managed as such. However, it is known that decreased BMD is related to childhood hypercalciuria and can affect adult bone health [39].

Several well-controlled clinical studies suggest that interferences in childhood bone mass acquisition may not affect adult bone mass after transient changes, possibly due to a homeostatic system that tends to return to normal following disturbances [96]. On the other hand, some studies emphasize that a persistent disturbing factor may compromise final bone mass in adulthood [96]. Thus, any continuous and persistent interference, such as untreated IH, especially with concomitant urolithiasis and/or hypocitraturia, may determine low BMD with increased risk of osteopenia, osteoporosis, and fractures in adulthood [13,97].

Recently, Perez-Suarez G et al. (2021) evaluated 34 hypercalciuric pediatric patients in a longitudinal study conducted over 20 years, with three bone densitometry evaluations in childhood, adolescence, and early adulthood. Both males and females showed increased bone mass over time, although the increase was statistically significant only in females. The authors attributed this to the positive effects of sex hormones and increased body mass. UCa and citrate excretion tended to decrease as patients reached adulthood, but lithogenic risk persisted. A positive correlation was found between BMI and BMD at all three evaluation points. They concluded that in patients with IH, BMD improves over time, particularly in females, likely due to body mass gain and reduced bone resorption (Table 2) [40].

Low BMD in children has been associated with an increased risk of fractures [98]. In a case-control study, Olney RC et al. found a significant association between frequent fractures and renal hypercalciuria in children with low BMD and without nephrolithiasis. In contrast, hypercalciuria in children without a history of fractures was primarily absorptive and did not affect BMD. The authors suggested that children with a history of frequent fractures should be evaluated for hypercalciuria as part of their assessment [98].

Overall, as shown by the aforementioned studies, assessment and treatment of hypercalciuria are essential in pediatric IH to prevent adverse outcomes.

Follow-up or monitoring

Monitoring or follow-up for IH typically involves regular assessments to manage the risk of nephrolithiasis and associated bone health issues, as well as to monitor treatment compliance and efficacy. Key aspects of monitoring include:

Urine Analysis

Regular 24-hour urine collections to measure calcium, phosphate, citrate, oxalate, potassium, and sodium.

Blood Tests

Periodic tests to check serum calcium, phosphate, urea, creatinine, other electrolytes, vitamin D, and iPTH. Biochemical bone markers can be measured easily and frequently to evaluate bone metabolism. Although all bone markers can assess bone disturbances, the International Osteoporosis Foundation and the International Federation of Clinical Chemistry recommend serum P1NP and CTX-I as the preferred markers for bone formation and resorption, respectively [65,70].

Ultrasound

Periodic ultrasound imaging to monitor for nephrolithiasis.

Bone Density Tests

DXA scans to monitor BMD, though these are rarely performed in children unless clinically indicated.

Dietary Assessment and Lifestyle Modifications

Evaluation of dietary intake of calcium, sodium, and protein, along with recommendations for adequate hydration and physical activity.

Medication Review

Monitoring the compliance, effectiveness, and side effects of medications, with adjustments as necessary.

Overall, the follow-up for IH should be individualized based on the patient’s symptoms, urinary parameters, and bone health status [39,97,23].

## Conclusions

Despite the limited studies in children and adolescents with IH, data show that one-third of the subjects present with lower BMD, even in the absence of nephrolithiasis, with the spine being most frequently affected. Several factors, such as the degree of hypercalciuria and the presence of calcium lithiasis, seem to affect the severity of bone disorders in children. Therefore, children with persistent IH should be evaluated for the level of calciuria, the presence of calcium lithiasis, as well as for possible bone metabolism abnormalities. Correction of IH appears to be important not only to prevent calcium lithiasis but also to improve bone metabolism.

Densitometry measurements help identify these children early and allow for timely treatment. Even though biochemical markers do not directly reflect bone mineral content, they provide valuable information on bone turnover. Hence, they can assist in monitoring treatment progress at shorter intervals than BMD measurements, albeit with several limitations in their use due to relatively low sensitivity and specificity, especially in pediatric populations.

Undoubtedly, there is a need for further investigation into the latest bone formation and resorption markers in children with IH, and for optimizing their sensitivity and specificity to detect subtle bone alterations.
